# Interdigitating Dendritic Cell Tumor of Submandibular Lymph Node: Case Report and Literature Review

**DOI:** 10.30699/IJP.2020.120698.2411

**Published:** 2020-12-21

**Authors:** Soroush Felezi, Anahita Nosrati, Mohammad Eslami Jouybari, Javane Jafarshad

**Affiliations:** 1 *Department of Pathology, Imam Khomeini Hospital, Mazandaran University of Medical Sciences, Sari, Iran*; 2 *Gastrointestinal Cancer Research Center, Mazandaran University of Medical Sciences, Sari, Iran*; 3 *Department of Pathology, Cancer Institute, Imam Khomeini Hospital Complex, Tehran University of Medical Sciences, Tehran, Iran*

**Keywords:** Dendritic cell, Interdigitating dendritic cell tumor, Lymph node, Sarcoma, Submandibular

## Abstract

Dendritic cells (DCs) are key arms of immune system, which act in antigen presenting processes, and are considered as a bridge between innate and adaptive immune responses. DCs are found in both lymphoid and non-lymphoid organs. They are called interdigitating dendritic cells (IDCs) in secondary lymphoid organs. IDCs lack lineage surface markers and are positive for S-100 and vimentin. IDC sarcoma (IDCS) is a very rare neoplasm, which mainly affects lymph nodes, though there are reports of extra-nodal involvement. IDCS is thought to have poor prognosis. Although there is no consensus on the treatment modalities, such options as radical surgery, chemotherapy, and radiotherapy are performed depending on severity and site of the lesion. In this study, we present a case of IDCS in a 53-year-old male with a history of several skin lesions and prior diagnoses of basal cell carcinoma (BCC), squamous cell carcinoma (SCC), and metatypical carcinoma (MTC).

## Introduction

Dendritic cells (DCs) are accessory cells of immune system and are capable to introduce antigens to T-cells. Interdigitating dendritic cells (IDCs) are a group of DCs which are mainly present in T-cell areas of secondary lymphoid organs like periarteriolar lymphoid sheath (PALS) of spleen and paracortex of the lymph nodes (1). Sarcoma of interdigitating dendritic cells (IDCs) is a rare malignancy mainly characterized by nodal involvement, and limitedly extra-nodal one. In nodal involvement, patients usually refer with lymphadenopathy; but in other organs involvement, the symptoms can manifest as abdominal pain, distension, skin lesions, and respiratory distress depending on the affected site. Fever, night sweats, and weight loss are observed in some patients too (1, 2). This malignancy can affect a wide range of ages, but usually the average age of patients is 50–60 years and there is a little male predominance (3). These cells have no specific markers but are positive for S-100 and vimentin and negative for B and T-cell markers (4). Although there is no consensus on the best treatment modality for IDCS due to its rarity, such treatment options as excision, chemotherapy, and radiation therapy have been applied with different outcomes (5). In this study, along with a literature review, we report a male patient diagnosed with IDCS with a previous history of basal cell carcinoma (BCC), squamous cell carcinoma (SCC), metatypical carcinoma (MTC), and non-surgical management.

Literature Review

We reviewed the case reports published from 2015 to 2019. To do so, we searched the Web of Science with the keyword "Interdigitating dendritic cell sarcoma", which resulted in the retrieval of 23 studies with 17 male and 14 female patients (age range: 22-82 years old). Studies with treatment strategies are shown in Table 1. According to the results of the studies, two patients died due to the complications of chemo-therapy, one patient died due to subsequent NK/T lymphoma which occurred two years after IDCS, and one patient died of pneumonia. Moreover, death was seen with all three modalities of surgery alone, chemotherapy, and/or radiation therapy. Most patients had lymph node involvement, but extra-nodal sites such as skin, gastrointestinal tract, respiratory, and central nervous system (CNS) involvement were also reported. 

**Table 1:** Published cases of interdigitating dendritic cell sarcoma (IDCS) from 2015 to 2019

Author	Gender/age of patient	Location or symptom of the tumor	Treatment modalities
HZ Chen* et al. *2019 (6)	Male/ 57-year-old	Right side of neck	Lymph node dissection, radiotherapy, chemotherapy (Nivolumab, doxorubicin, olaratumab, ipilimumab, nivolumab, Sirolimus, pazopanib)
Y Kajimoto* et al. *2019 (7)	Female/82-year-old	Right parotid gland	Parotidectomy, radiotherapy
C Zhao* et al. *(2019) (8)	Female/53-year-old	Spleen (Abdominal pain and mass)	Splenectomy, chemotherapy (ABVD, ifosfamide, oxaliplatin)
I Madabhavi* et al. *2018 (9)	Female/35-year-old	Right side of neck	chemotherapy (CHOP) Radiotherapy
HT Wang* et al. *2018 (10)	Male/64-year-old	Right groin	Chemotherapy (CHOP, etoposide, ABVD) Radiotherapy
Ayako Sakakibara* et al. *2018 (11)	Male/58-year-old	Left side of neck	Surgery Chemotherapy (CHOP) Radiotherapy
Guerra, F* et al. *2018 (12)	Female/23-year-old	jejunum (Abdominal bloating and discomfort)	Surgery
Ki Hwan Hong* et al. *2018 (13)	Female/59-year-old	Left side of neck	Excision
Tian Xue* et al. *(2018) (3)	Female/73-year-old	Cheek	Excision
	Female/72-year-old	Nasopharynx	Radiotherapy
	Male/61-year-old	Left side of neck	Excision
	Male/53-year-old	Submandibular lymph node	Excision
	Female/65-year-old	Left side of neck	None
	Female/46-year-old	Left side of neck	Excision, Radiotherapy
	Female/44-year-old	Left side of neck	Chemotherapy
Ninkovic, S* et al. *2017 (14)	Male/49-year-old	Neck lymphadenopathy	Excision CHOP chemotherapy gemcitabine/docetaxel
Magro, CM* et al. *2017 (15)	Male/55-year-old Male/47-year-old	peri-auricular mass Back nodule	Excision Radiotherapy Excision Chemotherapy (taxol and carboplatin)
Hwang, SY* et al. *2017 (16)	Female/75-year-old	Neck lymphadenopathy	Chemotherapy (ABVD)
Shi, F* et al. *2017 (2)	Male/40-year- old Female/62-year- old	Inguinal mass Respiratory symptoms	Chemotherapy (CHOPE DHAP) Chemotherapy (paclitaxel and nedaplati)
Jianguo Zhu* et al. *2017 (1)	Male/52-year-old	Pelvic mass (Abdominal distension)	Surgery
Catherine M. Nguyen and David Cassarino, 2016 (17)	Male/61-year-old	Multiple lesions on shoulder and thigh	chemotherapy (gemcitabine and docetaxel)
Valentina Lupato * et al. *2016 (18)	Male/81-year-old	Submandibular mass	dissection Radiotherapy
Gregor Hutter* et al. *2015 (19)	Male/39-year-old	Mild hemiparesis	Surgery
Shan, SJ* et al. *2015 (20)	Female/41-year-old	lesion on the shoulder	Excision
Di Liso, E* et al. *2015 (21)	Male/59 year-old	Axillary lymphadenopathy	Surgery Radiotherapy Chemotherapy (epirubicin, ifosfamide), BRAF inhibitor
O'Malley, DP* et al. *2015 (22)	Male/54-year-old man	Lymphadenopathy in groin	Excision Chemotherapy (R-CHOP, ifosfamide,carboplatin, etoposide)
C Kyogoku* et al. *2015 (5)	Male/62-year-old	Several skin lesions	Chemotherapy (ABVD)
Venkata K. Pokuri 2015 (23)	Male/81-year-old	enlarging and painless left neck mass	Dissection (two times)
Helbig, G* et al. *2015 (24)	Female/22-year-old	Neck lymphadenopathy	Excision Chemotherapy (ABVD)

## Case Presentation 

The patient was a 53-year-old man referred to the Oral and Maxillofacial Surgery Department of a teaching hospital due to a history of neck swelling for seven months. Ultrasound examination revealed an echo-free mass of 24.1x36.4 mm with regular shape and no adhesions to surrounding skin and at a depth of 3.9 mm from the skin of the left parotid region with possible diagnosis of benign mass or lymphadenopathy. The patient had photosensitivity and multiple skin lesions from childhood and at the age of 51, and he was diagnosed with BCC of the zygomatic region and lip, MTC of the frontal skin, and keratoacanthoma (KA)-like SCC of nose. Family history was unremarkable. He underwent radical lymph node dissection; in the histopathologic examination, a submandibular mass measured 4x2.5x1 cm was found, which was effaced by a malignant mesenchymal tumor with storiform pattern. Neoplastic cells were spindled-shaped with hyperchromatic, pleomorphic nuclei, vesicular chromatin, visible nucleoli, and eosinophilic cytoplasm. Mitosis (about 15 mitosis per 10 HPF), foci of hemorrhage, and necrosis were also noted. Based on mentioned findings, a primary diagnosis of metastatic malignant spindle cell tumor was made, but immunohistochemical (IHC) studies was suggested for a definitive diagnosis. Review of the hematoxylin and eosin (H&E)/trichrome-stained slides showed near total effacement of the node structure by fascicle and sheets of the tumor cells sparing follicles and sinuses. The tumoral cells were large and pleomorphic with abundant pale eosinophilic cytoplasm and bizarre, grooved nuclei showing some mitotic figures. Just a narrow rim of small lymphocytes was present in the outer aspect of the specimen. Considering the possible diagnosis, a wide IHC panel (Table 2) was performed and subsequently, the diagnosis of IDC sarcoma (tumor) was established. To detect whether the tumor is localized or has spread, contrast-enhanced spiral CT of the head and neck, chest, and abdominopelvic was ordered and no pathologic lesions and lymphadenopathy were observed. Therefore, no chemo-radiation was prescribed for the patient. Currently, ultrasound follow-up is on the process for the patient at the 3-month intervals ; the results has indicated no sign of recurrence as solid or cystic mass in neck tissue , and the patient is doing well. 

**Table 2:** Markers used in this IHC study

Positive														
Marker		S100		Vimentin			CD68		Desmin			CD45		
Pattern		Diffuse		Intense			Focal		scattered weak			in remnant lymphocytes		
Negative														
Marker	CD34		CD3		CD30	CD56		BCL2		HMB45	MNF116		P63	Melan A

## Discussion

IDCS is a rare hematologic malignancy with the highest prevalence reported in lymph nodes, though tumor can occur in extra-nodal sites such as lung, colon, and skin. For this reason, depending on the site of the lesion, different symptoms can take place; in head and neck areas the appearance of a mass or lymphadenopathy, in gastrointestinal tract and spleen, abdominal mass, distension, and pain (1, 8), and in lung and airway it can lead to respiratory symptoms (25). Due to fever, lymphadenopathy, and respiratory symptoms, suspicion of lymphoma can make diagnosis a challenging issue. Final diagnosis is based on histopathologic examination and IHC studies. Microscopic features of IDCS include spindled, ovoid, and epithelioid cells with eosinophilic cytoplasm and occasional prominent nucleoli, showing storiform, nodular, and fascicular growth patterns. Mitotic figures and necrosis may also be present (2, 3). Infiltration of the stroma with inflammatory cells like plasma cells and lymphocytes can also be observed. Tumoral cells are positive for S100, vimentin, CD68 (26) and are negative for CD1a, CD34, CD35, HMB45, Melan-A, CD21, and lineage markers for B- and T-cells (27). Differential diagnoses include non-hematologic (like melanoma, spindle cell SCC, and several mesenchymal neoplasms) and hematologic neoplasms (follicular DC sarcoma, Langerhans cell histiocytosis, and fibroblastic reticular cell tumor). There is still no consensus on the choice of treatment for this malignancy. However, considering the stage of the disease and whether the lesion is localized, or it has spread, different modalities including surgery, chemotherapy, and radiation are applied. Chemotherapies usually contain CHOP regimen (Cyclophosphamide, Doxorubicin, Vincristine, and Prednisone), ABVD regimen (Doxorubicin, Bleomycin, Vinblastine, and Dacarbazine), DHAP regimen (Cisplatin, Cytarabine, and Dexamethasone), taxol, carboplatin, ifosfamide, and oxaliplatin (2, 8, 15). We chose surgical dissection and carefully monitored the patient because the lesion was localized. There are reports of recurrence of tumor and death despite chemotherapy. Appropriate response to treatment with ABVD regimen has also been observed. Potential side effects should also be considered when choosing chemotherapy, as chemotherapy-induced liver injury which can be fatal. 

The etiology of IDCS is not yet fully understood due to its rarity. BRAF mutations (a kinase involved in cell signaling) which frequently occur in some hematologic neoplasms like Langerhans cell histiocytosis (LCH) and hairy cell leukemia (HCL) are found in IDCS too (21, 28). Bailleux* et al. *(29) applied initial low dose BRAF inhibitor (Vemurafenib) to treat a male patient with relapsed HCL, which led to complete remission (30). Eight months later and due to the relapsed disease, vemurafenib was re-administrated and complete hematologic response was achieved (29). This treatment was also prescribed by Di Liso* et al. *for their IDCS patient and resulted in tumor shrinkage but toxicity was also noted (21). Programmed death-ligand 1 (PD-L1) is an immune inhibitory protein which suppresses tumor specific immune responses by binding to PD1 on activated T cells. Sakakibara* et al. *showed membranous and cytoplasmic expression of PD-L1 on IDCS cells of a patient who died from a progressive disease. This is important in terms of how tumoral cells evade detection by the immune cells and potential therapeutic targets (11).

An interesting study performed whole exome sequencing (WES) on the blood and tumor specimens of an IDCS patient; the results showed that alteration in DNA polymerase theta (POLQ) and folliculin interacting protein 1 (FNIP1) genes may be involved in malignant potential of IDCs (13). IDCS has also been developed in patients who received organ transplantation; so, the question is raised if immunosuppressive agents could be as causes of IDCS (3). Although medical history in several patients is unremarkable, presence of a previous or simultaneous malignancy is observed in some reports. The present patient had a history of multiple skin cancers. Rosenberg* et al. *presented a female patient with BCC of back skin (31), and Lee* et al. *reported an IDCS patient with an unknown type of skin cancer (32). So, it is unclear whether previous therapies for cancer have triggered IDCS development or not. There are also observations of increased risk of other malignancies in the following years in IDCS patients. Therefore, it is important to monitor patients even after the malignancy is treated. Finally, our patient responded well to surgery due to the localization of the lesion, but given that many patients have been reported to present with progressive disease and die of IDCS, a chemo-radiation therapy should be considered if the disease is multifocal and other organs are involved.
